# Clinical evaluation of sinus bone graft in patients with mucous retention cyst

**DOI:** 10.1186/s40902-016-0081-1

**Published:** 2016-09-25

**Authors:** Seong-Beom Kim, Pil-Young Yun, Young-Kyun Kim

**Affiliations:** 1 Department of Oral and Maxillofacial Surgery, Section of Dentistry, Seoul National University Bundang Hospital, 300 Gumi-dong, Bundang-gu, Seongnam, Gyunggi-do South Korea; 2 Department of Dentistry & Dental Research Institute, School of Dentistry, Seoul National University, Seoul, South Korea

**Keywords:** Maxillary sinus, Sinus augmentation, Mucous retention cyst

## Abstract

**Background:**

Mucous retention cyst refers to a cyst made by expansion due to the blockage of the salivary gland near the maxillary sinus, and it is surrounded by epithelial cells. Most of them are small; therefore, they cannot be found well and are frequently with antral polyp. The aim of this study was to evaluate the clinical prognosis of sinus bone graft in patients with mucous retention cyst.

**Methods:**

This study was performed retrospectively on 23 patients who had sinus bone graft. Group 1 was 8 patients (10 sinuses) who had a mucous retention cyst, and group 2 was 15 patients (17 sinuses) who had no pathologic history about the maxillary sinus. For these patients, sinus bone graft was performed using the lateral approach technique. The total 51 implants were placed 6.22 weeks on the average after sinus bone graft. Sinus membrane perforation during operation, postoperative complications, marginal bone loss after restorative function, implant success rate, and survival rate were analyzed.

**Results:**

There was no complication in group 1, and there were three complications in group 2. In group 2, two cases of implants failed. The types of postoperative complications consisted of two minor infections and one wound dehiscence. Two implants of total 51 implants were removed, and the survival rate of implants was 96.08 % (group 1 100 %, group 2 93.5 %). The total success rate of implants was 92.2 % (group 1 95 %, group 2 90.3 %).

**Conclusions:**

The clinical prognosis was not affected by the presence of mucous retention cyst.

## Background

There are many difficulties in the placement of implants of the maxilla because of deficient residual alveolar bone. In such cases, sinus bone graft is performed. The success rate of sinus bone graft is high, and the technique is predictable, thus forming the basis of successful placement of implants [1, 2]. The shape of the maxillary sinus is like a pyramid, and it is connected with the nasal cavity and paranasal sinuses. Therefore, if the physiological status of the maxillary sinus is not normal, the possibility of complications after bone graft can increase [3, 4].

The cyst formed at the maxillary sinus is usually found in the radiographic view by chance, and it is divided into four types: pseudocysts, mucoceles, postoperative maxillary cysts, and mucous retention cysts.

Pseudocyst is the thickening of the sinus membrane due to the local retention of inflammatory exudation. Occurring as a faintly dome-shaped radiopaque lesion at the floor of the maxillary sinus in the radiographic view, it is referred to as “pseudo” cyst because it is actually not surrounded by epithelial cells. It is not harmful, and treatment is not needed. Sometimes, the term is confused with mucocele, but it has a more destructive character [5].

Mucocele surrounds the wall of the maxillary sinus and has an aggressive, destructive character. It is surrounded by epithelial cells and is filled with mucous fluid, occurring mostly when the drainage of mucus is poor and in case of lack of patency of the natural ostium [5].

Postoperative maxillary cyst occurs after the operation related to the maxillary sinus, such as a Caldwell-Luc operation. It appears as a unilocular radiopaque lesion with clear margin, surrounded by respiratory-type epithelial cells. It seems like a postoperative change that mucosal tissue was entrapped into the wound after closure and healing of the maxillary sinus.

Mucous retention cyst refers to a cyst made by expansion due to the blockage of the salivary gland near the maxillary sinus, and it is surrounded by epithelial cells. Most of them are small; therefore, they cannot be found well and are frequently with antral polyp [3–6].

In this study, we evaluated the clinical prognosis and postoperative complications of the implants when sinus bone graft is performed on patients with mucous retention cyst.

## Methods

This study was performed retrospectively on patients with mucous retention and who visited and had dental implants with sinus augmentation and bone graft by a lateral approach at the Department of Dentistry, Seoul National University Bundang Hospital, from January 2008 to December 2010. The average age was 53.43 years (26~74 years). The patients with pathology of the maxillary sinus and those who were treated for chronic maxillary sinusitis by an otolaryngologist were excluded. The patients in this study were 23 people (males 15, females 8) and were analyzed through the medical record and radiographs. They were classified into two groups. Group 1 (*n* = 8), test group, included the patients who had a mucous retention cyst before the implant treatment, and group 2 (*n* = 15), control group, included those who had a healthy maxillary sinus. Seven patients had systemic diseases like hypertension and diabetes mellitus and were controlled well (Table 1).

**Table 1:** Case distribution

Total (*n* = 23)		Group 1 (mucous retention cyst)		Group 2 (normal sinus)	
Males	15	7		8	
Females	8	1		7	
Average age		53.43 (26 to 74 years)			
Systemic diseases	n/s 16	HT 4	DM 1	HBV 1	Leukemia 1

n/s: non-specific

The diagnosis of mucous retention cyst was done through the radiographs and aspiration of mucus from the maxillary sinus membrane during the operation. The clinical and radiological character in the diagnosis of the disease is as follows [6]:

1. Most of the mucous retention cysts are clinically asymptomatic and are found in the radiographic view by accident.
2. They are mostly observed as dome-shaped radiopaque lesions on the floor of the maxillary sinus in the radiographic view.
3. Because they originate in the outside of the maxillary bone, the margin of the lesion is not surrounded by the radiopaque line of cortical bone, unlike dentigerous cysts [7].

### Surgical technique

**Fig. 1:**
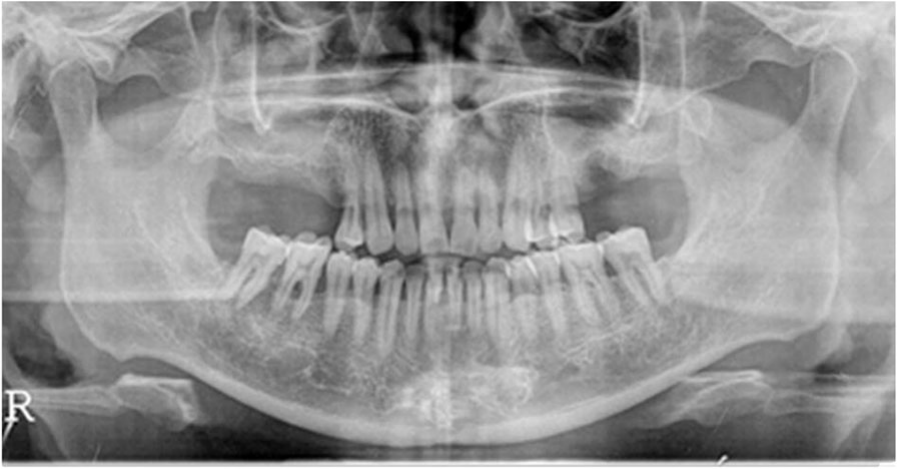
Preoperative panoramic radiograph of 46-year male patient. Residual bone height is about 2–3 mm, and sinus radiopacity is observed in the right maxilla

**Fig. 2:**
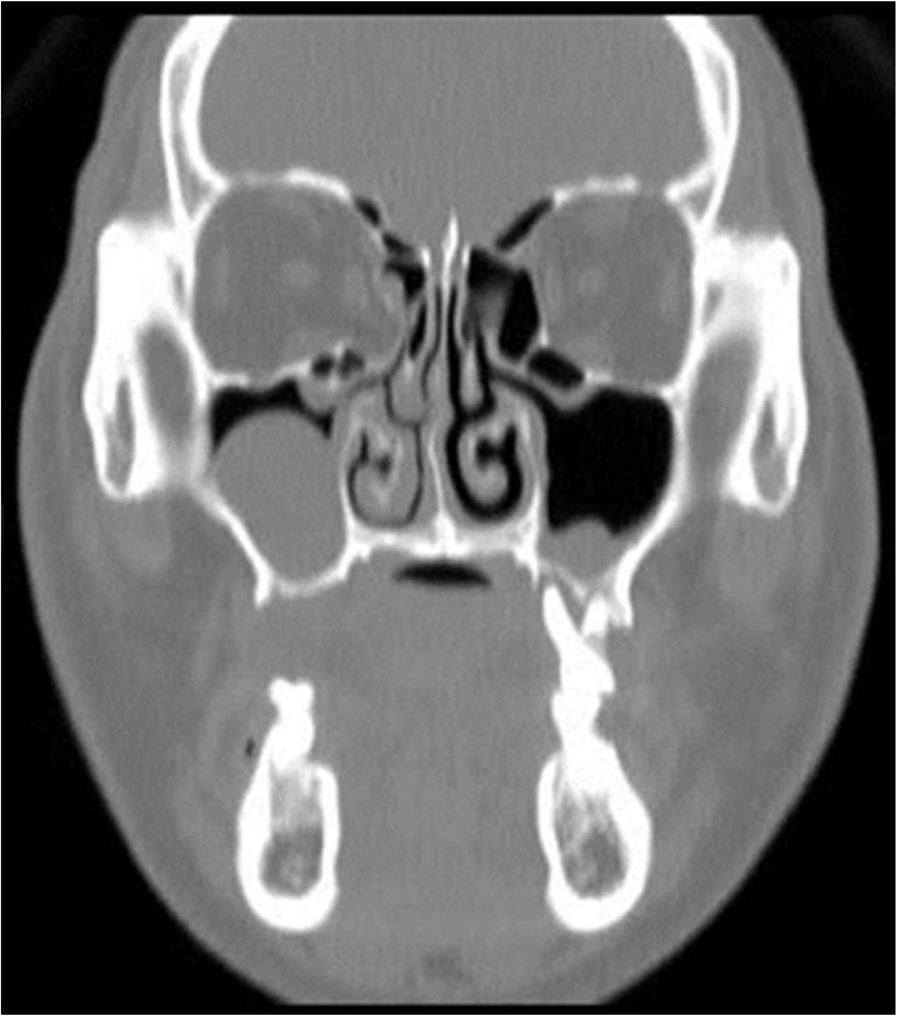
Preoperative PNS CT view. Dome-shaped radiopacity is observed in the right maxillary sinus

**Fig. 3:**
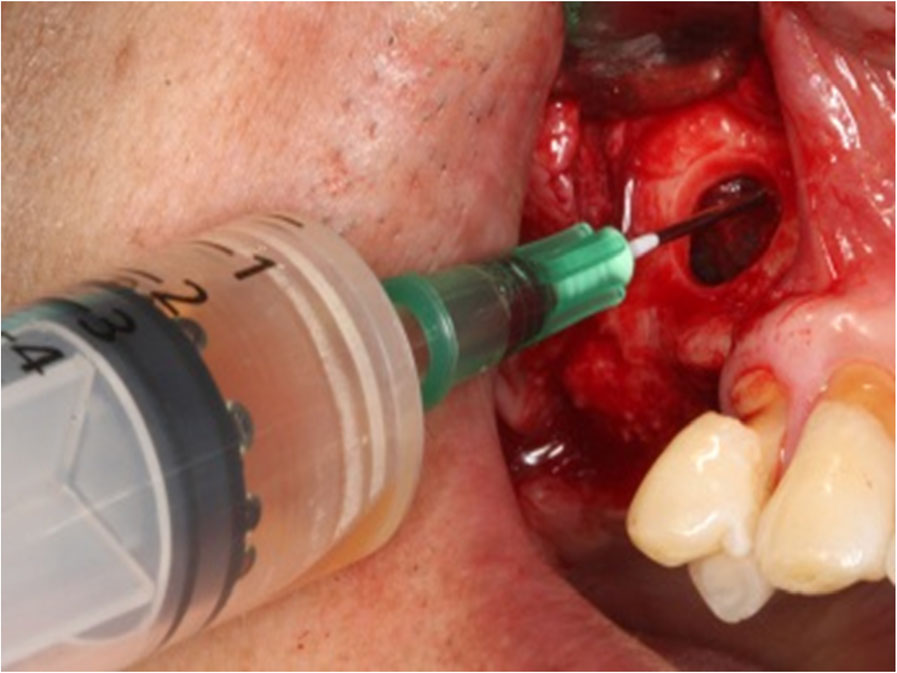
Crestal incision was done on the right maxillary alveolar ridge, with the flap elevated. Mucous fluid was aspirated by opening the lateral wall of the maxillary sinus

**Fig. 4:**
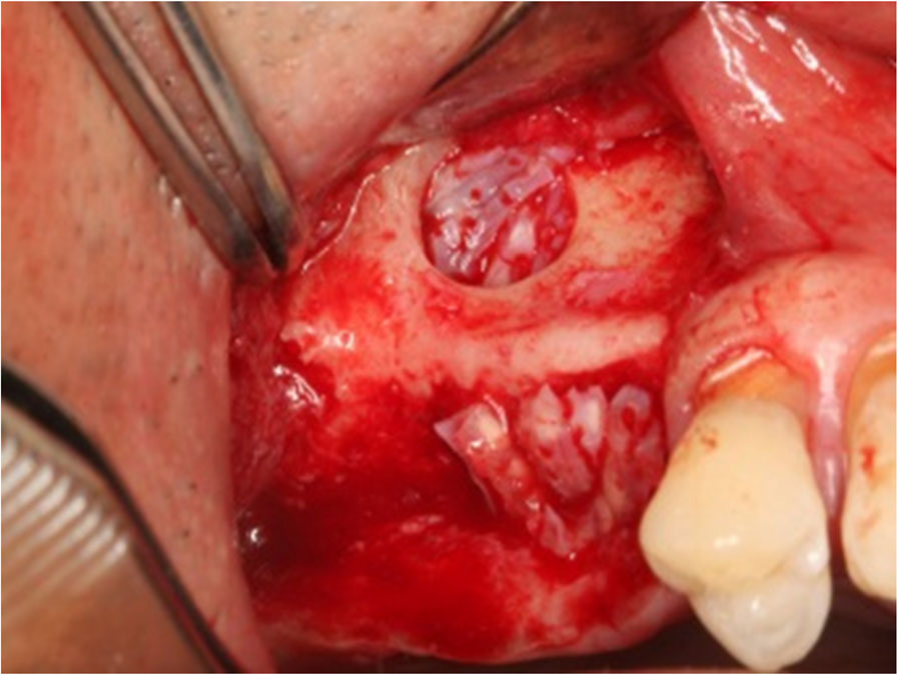
Sinus bone graft was performed after sinus membrane elevation

**Fig. 5:**
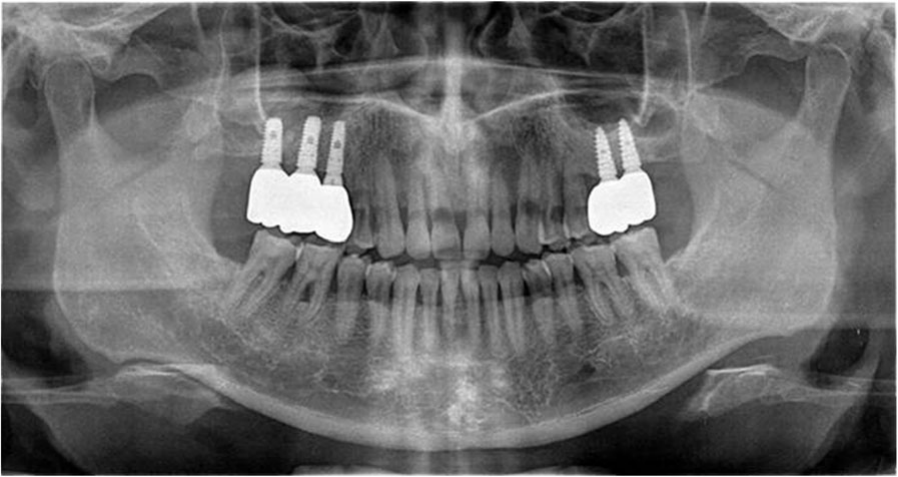
Panoramic radiograph 3 years and 9 months after sinus bone graft. There were no postoperative complications

Preoperative gargling by 0.2 % chlorhexidine solution was performed before local anesthesia. The mucoperiosteal flap was elevated after crestal and vertical releasing incisions at the edentulous site. A lateral sinus window was formed and removed by surgical drill, and the sinus membrane was elevated. Aspiration was done by inserting a 21-gauge needle into the maxillary sinus membrane in cases of mucous retention cysts. After that, the membrane was lifted, and bone graft was done following the aspiration of mucus. If the sinus membrane was punctured broadly, it was covered by a collagen membrane. The maxillary sinus was augmented using the prepared bone graft material, the bony window was put back to the original position, and the wound was closed (Figs. 1, 2, 3, 4, and 5). Augmentin 625-mg tab (GlaxoSmithKline, UK) as antibiotics, Somalgen 370-mg tab (Kunwha Pharmaceutical Co., Republic of Korea) as anti-inflammatory and analgesic drug, and Methylon 4-mg tab (Kunwha Pharmaceutical Co., Republic of Korea) as steroid drug were prescribed. 0. 1 % chlorhexidine soln 100 ml (Hexamedine, Bukwang Pharm, Ansan, Korea) gargling was done three times a day, with the sutures removed after 10 days.

A total of 51 implants (group 1 20, group 2 31) were placed, mostly with average waiting time of 6.22 weeks after sinus bone graft. Note, however, that implants were placed immediately after sinus bone graft for 13 cases (the period until the implants were placed was calculated by counting the healing period as 0 day in the direct placement method). The restoration was connected 29.41 weeks on the average following implant placement and after the radiographic view was taken. It was observed for an average of 43.29 months at an interval of 3~6 months after connection with the restoration. Through medical record, radiographs, sinus membrane perforation during operation, postoperative complications, marginal bone loss after restorative function, implant success rate, and survival rate were analyzed.

The periapical views were taken vertically to the length of the implants to calculate and to compare the values of marginal bone loss. The distance from the platform of an implant to the first site meeting to crestal bone was measured, and the real distance was obtained by applying the enlargement ratio of radiographs. The enlargement ratio was found by a proportion between the real length of an implant and the length shown at the radiograph. After that, the average of change of mesial and distal marginal bone loss was analyzed.

The implant success criteria were as follows [8]:

1. Absence of continuous or irreversible pain, discomfort, and/or paresthesia
2. Absence of recurring peri-implantitis with abscess
3. Absence of mobility
4. Absence of radiolucent lesion(s) around the implant
5. Marginal bone loss of less than 1 mm in the first year

The analysis of marginal bone loss around the implants was performed using SPSS 17.0 (Statistical Package for the Social Sciences; SPSS, Inc., Chicago, IL). The Mann-Whitney *U* test was performed when these assumptions were not fulfilled. *p* < .05 was considered statistically significant.

## Results

In five cases of group 1, large perforations were found on the sinus membrane and closed by a collagen membrane. There were no perforations in group 2. After the operation, three patients had complications. The postoperative complications were only found in group 2. There were no surgical complications in group 1 with mucous retention cysts. In postoperative complications, the infections were two cases and the wound dehiscence was one case. The osseointegration failed in two implants. Most of the complications subsided by wound dressing, antibiotic medication, incision and drainage, and implant removal and replacement.

After implant installation, the restorations were connected. After 1 year of restorative function, the marginal bone loss around the implant was measured. With the exception of the implants of no radiographs or which failed during observation, the marginal bone loss of 11 cases of group 1 and 22 cases of group 2 was measured. The average of marginal bone loss of group 1 was 0.10 ± 0.40 mm. That of group 2 was 0.06 ± 0.24 mm. More bone loss was found in group 2 than in group 1, but it was not significant statistically(*p* = .919).

In this study, 2 implants of total 51 implants were removed, and the survival rate of implants was 96.08 % (group 1 100 %, group 2 93.5 %). There was a case that had more marginal bone loss than 1 mm in 1-year restoration in each group. Therefore, the total success rate was 92.2 % (group 1 95 %, group 2 90.3 %) (Table 2).

**Table 2:** Postoperative complications, survival, and success rates of implants

	No. of sinuses (27)	No. of implants	Post OP complications	Implant osseointegration failures	Survival rate (%)	Success rate (%)
Group 1	10	20	0	0	100	95
Group 2	17	31	3	2	93.5	90.3

## Discussion

Placing an implant in the maxillary molar area is frequently very difficult due to the limitation in terms of the quality and quantity of the alveolar bone of the maxilla [9]. Therefore, bone graft is usually needed for the placement of implants in the molar area of the maxilla. Sinus bone graft is a predictable technique as reported in many previous articles [10, 11]. For many decades, it has secured a safe basis for the placement of implants [12]. According to some articles, however, sinus bone graft has been contraindicated when there is some sort of cyst in the maxillary sinus [13]. Therefore, the authors claimed that sinus bone graft should be performed only when there is no cyst in the maxillary sinus. On the other hand, some articles report that a cyst in the maxillary sinus does not affect the prognosis of sinus bone graft [14]. For the study of Maiorana et al. involving 10 patients with mucosal cyst, the implants were placed after sinus bone graft, and the implants osseointegrated successfully for 28 months during observation. They reported a 100 % survival rate [15]. In this study, sinus bone graft and implant placement were performed on 23 patients (males 15, females 8) with/without mucous retention cyst. During the follow-up period of 43.29 months on the average after prosthetic function, total survival rate of implants was 96.08 %, and total success rate was 92.2 %. However, the survival rate was 100 %, and success rate was 95 % in group 1. In the cases of mucous retention cyst, the mucus of the maxillary sinus was aspirated prior to sinus membrane elevation. This could decompress internal pressure, reduce the size of the cyst, and decrease the possibility of laceration of the Schneiderian membrane during sinus membrane elevation. Therefore, the sinus membrane has to be elevated carefully from the bony floor using antral curette [15]. In this study, however, there were five cases of eight cases in which the sinus membrane was perforated and closed by a collagen membrane and the sinus augmentation was done successfully. In diagnosis of mucous retention cyst, there is limitation using radiographs. The size of the lesion of mucous retention cyst was not large enough to be found in radiographs, and the mucus should be aspirated to confirm the diagnosis. To rule out the POMC, the patients were asked whether they had a surgery related to the maxillary sinus, and to rule out pseudocyst, it was checked whether the epithelial cells surround the lesion. And also, mucocele was ruled out through the character and size of the lesion. Mucoceles usually have a destructive character and large size of the lesion.

When implants are placed after sinus bone graft using the lateral approach technique, the survival rate is 91.8 % (61. 7~100 %) on the average depending on the article [16]. In this study, the total survival rate of implants was 96.8 % which is similar to previous articles, but the survival rate of group 1 was 100 %. The reason maxillary sinusitis occurs after sinus bone graft is related to the dysfunction of the drainage of mucus. Patency of the ostium is the basis of assurance of physiological mucus drainage, decreasing the possibility of postoperative maxillary sinusitis. Therefore, if the patient has maxillary sinusitis before, it is important to secure the patency of the natural ostium [14]. In this study, the treatment of mucous retention cyst was immediately done during the sinus bone graft, exposing the sinus cavity by the lateral window approach and doing aspiration. As a result, some cases had postoperative complications, but all of them subsided after additional antibiotics and conservative treatment. The implants placed after the treatment showed adequate clinical prognosis.

## Conclusions

Based on this finding, it is possible to place implants immediately after sinus bone graft on patients with inactive sinus pathologic condition such as mucous retention cysts. This study included only eight patients (10 sinuses), which was a small group; with more patients, however, the results will be more meaningful. In conclusion, if adequate treatment is performed, good clinical prognosis can be expected from the placement of implants after sinus bone graft on patients with mucous retention cyst.
